# *Mycobacterium abscessus*: Shapeshifter of the Mycobacterial World

**DOI:** 10.3389/fmicb.2018.02642

**Published:** 2018-11-01

**Authors:** Keenan Ryan, Thomas F. Byrd

**Affiliations:** ^1^Department of Pharmacy, University of New Mexico Hospital, Albuquerque, NM, United States; ^2^Department of Medicine, The University of New Mexico School of Medicine, Albuquerque, NM, United States

**Keywords:** glycopeptidolipid, toll-like receptor 2, cystic fibrosis, bronchiectasis, serpentine cording, fibroblasts

## Abstract

In this review we will focus on unique aspects of *Mycobacterium abscessus* (*MABS*) which we feel earn it the designation of “shapeshifter of the mycobacterial world.” We will review its emergence as a distinct species, the recognition and description of *MABS* subspecies which are only now being clearly defined in terms of pathogenicity, its ability to exist in different forms favoring a saprophytic lifestyle or one more suitable to invasion of mammalian hosts, as well as current challenges in terms of antimicrobial therapy and future directions for research. One can see in the various phases of *MABS*, a species transitioning from a free living saprophyte to a host-adapted pathogen.

## Evolving Nomenclature

*Mycobacterium abscessus* (*MABS*), a rapidly growing non-tuberculous mycobacterium (NTM) (Howard and Byrd, 2000) is an emerging pathogen worldwide. Perhaps the earliest case of *MABS* was reported in 1951 and described infection which occurred in the setting of traumatic knee injury. This infection was characterized by “subcutaneous, abscess-like lesions with a peripherally tuberculoid structure.” The unique characteristics of this mycobacterium prompted the investigators to propose it as a new species, *Mycobacterium abscessus* (Moore and Frerichs, 1953). In more recent times, *MABS* was considered a subspecies of *Mycobacterium chelonae* until 1992 when genetic analysis demonstrated that it was a distinct species and it was elevated to species status as *MABS* (Kusunoki and Ezaki, 1992). This resulted in the realization that most prior reports of *M. chelonae* lung infection were likely a mischaracterization of actual *MABS* lung infection as it is now recognized that pulmonary infection is a common clinical manifestation of *MABS* whereas it is rarely caused by *M. chelonae.* In recent years, *MABS* has been further characterized into three distinct subspecies; *MABS* subspecies *abscessus*, *MABS* subspecies *bolletii* and *MABS* subspecies *massiliense* (Adekambi et al., 2017). They differ in terms of drug susceptibility, and may have differences related to transmissibility as well which will be discussed (Tan et al., 2017). Genomic analysis of *MABS* reveals evidence of horizontal gene transfer (Howard et al., 2002). In addition to genes associated with mycobacterial virulence, genes with similar function to those found in *Pseudomonas aeruginosa* and *Burkholderia cepacia*, pathogens commonly found in the lungs of cystic fibrosis (CF) patients, have also been identified (Ripoll et al., 2009). Thus *MABS* can be thought of as a pathogen uniquely adapted to different niches within the host lung environment.

## Epidemiology and Emergence as a Pathogen

The incidence of NTM infection is increasing in the United States (Prevots et al., 2010) and worldwide (Prevots and Marras, 2015). Pulmonary infection is the most common clinicalpresentation, however, extrapulmonary infection either due to direct inoculation into the skin or due to disseminated disease, often in association with disease modifying anti-TNFα therapy, is also being recognized with increased frequency (Winthrop et al., 2009). In the United States, *Mycobacterium avium* complex (*MAC*) is the most common NTM clinical pulmonary isolate followed by *MABS* (Prevots and Marras, 2015). It has also been reported that there are geographic differences in the types of NTM isolates in the United States. *MAC* is the most frequent and predominant isolate in the South and Northeast with proportionally higher rates of isolation of *MABS*, *M. chelonae*, *M. fortuitum*, and *M. kansasii* in the Western United States (Spaulding et al., 2017). It is noteworthy that in parts of Asia *MABS* is the predominant pulmonary NTM pathogen (Umrao et al., 2016; Nagano et al., 2017; Lim et al., 2018). Furthermore, limited epidemiologic data suggests that there may be a relative genetic susceptibility to *MABS* infection in certain Asian populations (Adjemian et al., 2017).

Perhaps what is most disconcerting about *MABS* from an epidemiologic standpoint is its unique ability among NTM to cause outbreaks of infection over wide geographic regions without linkage to a single or specific point source. The best described instance of this relates to a study of post-surgical wound infections with *MABS* subspecies *massiliens*e in Brazil. Using genomic analysis it was found that a recently emergent single clone caused multiple outbreaks of post-surgical wound infections. The organism spread throughout Brazil and persisted in hospital environments. Evidence was also provided that there is a loss of genetic material from this lineage raising the possibility that it is undergoing reductive evolution as it adapts to its new niche in the hospital environment (Everall et al., 2017). It is noteworthy that this isolate is genetically related to another *MABS* subspecies *massillense* clone that has been reported to be circulating among CF treatment centers throughout the world. There is evidence that acquisition of this strain by CF patients may be associated with worse clinical outcomes. Data from cell culture and animal infection experiments provide evidence that this clone may be more virulent in comparison to unclustered *MABS* subspecies *abscessus* isolates. Review of this data shows that although the observed differences are statistically significant, they are small (Bryant et al., 2016), and whether they have clinical relevance in terms of virulence remains unclear. Nonetheless these studies raise the disconcerting possibility that evolutionary changes affecting transmissibility and adaptation to mammalian hosts could lead to further spread of *MABS* into the susceptible general population of patients with abnormal lung airways and/or states of immunosuppression (Winthrop et al., 2009). The possible mechanisms responsible for spread of *MABS* include fomites, aerosolized airway secretions and contaminated hospital and municipal tap water (Baker et al., 2017; Donohue, 2018). One study has reported an association between warm humid climates and high atmospheric vapor pressure with the prevalence of NTM infection (Prevots and Marras, 2015). This raises the question of whether climate change is contributing to the rising incidence of NTM infection.

## Clinical Infection

The clinical spectrum of *MABS* and other rapidly growing mycobacteria has been well-described and broadly categorized as pulmonary and extrapulmonary disease (Howard and Byrd, 2000). *MABS* extrapulmonary infection can involve a variety of sites, most commonly the skin. It can occur via dissemination in immunosuppressed patients (Scholze et al., 2005), often from an occult source, or from direct inoculation, either iatrogenically [for example, contaminated acupuncture needles, injection solutions, and surgical procedures (Ryu et al., 2005; Yuan et al., 2009; Schnabel et al., 2016)], or as a result of wound contamination in the setting of trauma (Petrini et al., 2006). Infection of many anatomic sites has been reported including the eye, bone, joint, and central nervous system (Chu et al., 2015; Fukui et al., 2015; Baidya et al., 2016; Jeong et al., 2017). Extrapulmonary infection is generally responsive to treatment with antibiotics to which the isolate is susceptible with adjunctive surgical debridement where indicated.

*MABS* was first recognized as a cause of chronic pulmonary infection in 1993. The majority of pulmonary infections occur in older adults often with no history of cigarette smoking who are otherwise healthy, but who have underlying lung airway abnormalities (Griffith et al., 1993). The clinical presentation is indistinguishable from lung infection caused by *MAC*. Unlike *MAC*, however, effective antibiotic treatment options are limited with many patients requiring a combination of medical and surgical intervention for cure (Jarand et al., 2011). In patients with CF, *MABS* pulmonary infection is an important cause of morbidity and mortality, and is a relative contraindication to lung transplantation (Dorgan and Hadjiliadis, 2014). It is important to note that immune dysregulation as a result of mutations in the *CF Transmembrane Conductance Regulator gene* (*CFTR*) contributes to the inflammatory phenotype in CF lung disease, and may result in a pathogenic process that differs from that seen in otherwise healthy individuals who have *MABS* infection and abnormal lung airways (Rieber et al., 2014). One clinically apparent difference is that *MABS* chronic colonization of the lung by smooth variants as well as by rough invasive variants is a cause of morbidity in CF patients (Bryant et al., 2016). In otherwise healthy patients with abnormal lung airways *MABS* colonization is more likely to be transient.

A common characteristic of patients with *MABS* pulmonary infection is bronchiectasis. An obvious question is whether bronchiectasis is the end result of infection with *MABS* or a predisposing factor for *MABS* infection? An experiment of nature provides evidence that bronchiectasis *per se* predisposes to NTM infection in the absence of preceding inflammation. Patients with the genetic disorder known as primary ciliary dyskinesia have a loss of normal ciliary structure and function leading to development of bronchiectasis which may occur in the absence of antecedent inflammation. These individuals are prone to recurrent oto-sino-pulmonary infections. In addition to lung infection with pyogenic bacteria such as *P. aeruginosa* and *Staphylococcus aureus*, these patients are also susceptible to colonization and invasive infection with *MABS* and *MAC* (Noone et al., 2004). The other lung airway disease which predisposes to colonization and infection with NTM such as *MABS* and *MAC* is chronic obstructive pulmonary disease (COPD) (Billinger, 2009). Large upper lobe bullous cavities in the lung are a predisposing factor to NTM infection in these patients. It is also noteworthy that bronchiectasis is also present in a high percentage of patients diagnosed with COPD (Patel et al., 2004; Martinez-Garcia et al., 2013) suggesting that the presence of bronchiectasis may be an important factor in *MABS* and *MAC* disease pathogenesis in these patients as well.

## Pathogenesis: the Importance of Rough and Smooth Colony Phenotypes

Microdroplet nuclei aerosolized after an individual with pulmonary tuberculosis coughs, with subsequent inhalation by an uninfected host, is the mode of person to person spread of *M. tuberculosis*. In contrast, epidemiologic studies have not demonstrated this to be an efficient mechanism for acquisition of pulmonary *MABS* infection although there is experimental evidence that long lived infected aerosols generated by a coughing patient may contain respiratable bacteria (Bryant et al., 2016). Respiratable bacteria could also result from other sources of aerosols contaminated with *MABS*, for example colonized showerheads. Fomites have also been implicated in transmission of an outbreak strain (Bryant et al., 2016). It would seem unlikely that bacteria adherent to a fomite could be easily re-aerosolized. This mechanism of transmission could involve contact transfer from the fomite to the mouth with subsequent colonization of the oropharynx. Microaspiration of bacteria into the lung would lead to colonization of the lung airways and/or invasive lung infection (Thomson et al., 2007).

**FIGURE 1:**
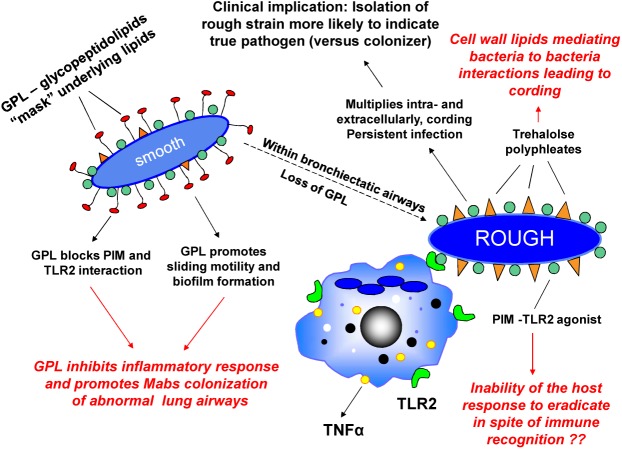
Environmental *Mycobacterium abscessus* (*MABS*) isolates have the smooth colony morphotype and express glycopeptidolipid (GPL). GPL in the outermost aspect of the cell wall “masks” underlying glycosylated lipoproteins such as phosphatidyl-myo-inositol mannosides (PIMs) involved in immune recognition and blocks the bacterial cell–cell interaction of lipids such trehalose polyphleates which may play a role in clumping and cord formation. By preventing *MABS* from being recognized by innate immune surveillance mechanisms and promoting biofilm formation, GPL facilitates colonization of bronchiectatic lung airways. After colonization, spontaneous or temperature sensitive loss of GPL is associated with “unmasking” of these molecules. This leads to recognition by TLR2 on macrophages and respiratory epithelial cells resulting in release of the proinflammatory cytokines TNFα (from macrophages) and IL-8 (from respiratory epithelial cells). Rough variants acquire a virulent phenotype characterized by the ability to grow in serpentine cords and cause macrophage apoptosis leading to rapid cell–cell spread and propagation of infection. Isolation of *MABS* rough variants from the sputum is associated with progressive lung infection.

We first described rough and smooth colony phenotypes of *MABS* which arose from a single parental strain, and demonstrated that the rough variant persists in the lungs of SCID mice, replicates in macrophages, and forms corded, invasive microcolonies in fibroblast monolayers. The smooth variant demonstrated none of these characteristics (Byrd and Lyons, 1999). We then identified glycopeptidolipid (GPL) in the cell wall of the smooth variant as being responsible for the smooth colony phenotype and for the ability of the smooth strain to exhibit sliding motility and biofilm formation (Howard et al., 2006; Nessar et al., 2011). Importantly, we demonstrated that a mutant which lacks GPL and exhibits the rough phenotype can spontaneously arise from the smooth phenotype and regain virulence characteristics (Howard et al., 2006). We showed by light and electron microscopy (Byrd and Lyons, 1999; Howard et al., 2006), and in a later publication using scanning electron microscopy (Sanchez-Chardi et al., 2011), that the rough phenotype exhibits cording, a property associated with virulence in *M. tuberculosis*. These observations were replicated by others, and the ability of spontaneous rough mutants to arise in infected mice from initial infecting *MABS* smooth variants was subsequently demonstrated (Catherinot et al., 2007; De Groote et al., 2014). The clinical relevance of these observations is supported by studies indicating that the majority of clinical isolates from individuals with chronic lung disease exhibit the rough phenotype whereas environmental contaminants and isolates from wounds exhibit the smooth phenotype (Jonsson et al., 2007). A study which longitudinally characterized *MABS* isolates from ten CF and three non-CF patients over a 10 year period found that a switch from smooth to rough colony morphology was observed in 6 of the patients during the course of long-term infection and was associated with increased severity of clinical symptoms (Kreutzfeldt et al., 2013). In addition, it has been documented that deterioration of lung function in a *MABS*-infected CF patient was associated with conversion of a sputum isolates from the smooth phenotype to an isogenic rough phenotype (Catherinot et al., 2009). These observations are consistent with our hypothesis that *MABS* initially gains entry to the lung as a GPL-expressing smooth variant which colonizes abnormal lung airways, and that spontaneous loss of GPL expression leads to a virulent phenotype capable of causing inflammation and invasive lung disease (Howard et al., 2006; Rhoades et al., 2009). In support of this pathogenic mechanism, we have provided evidence that *MABS* GPL is immunologically inert and that loss of GPL unmasks underlying *MABS* cell wall molecules such phosphatidyl-myo-inositol mannosides (PIMs) which are recognized by toll like receptor 2 (TLR2) on macrophages and epithelial cells thereby initiating an inflammatory response. Establishment of a smooth strain in the lungs of individuals with normal lung airways is likely prevented by the normal lung mucociliary clearance mechanism. This prevents establishment of lung airway biofilm from which invasive rough variants which have lost GPL can emerge. In patients with abnormal lung airways and diminished mucociliary clearance, *MABS* smooth variants have been demonstrated to establish biofilm in lung airways (Qvist et al., 2015). In CF patients, heavy colonization of lung airways with smooth variants in the absence of lung invasion is also likely to contribute to morbidity. Evidence indicates that genetic mutation involving a gene coding for one of the MmpL proteins involved in the biosynthesis of the *MABS* cell envelope is responsible for the smooth to rough transition in one *MABS* strain (Bernut et al., 2016b). However, we have presented evidence that in some strains of *MABS*, expression of GPL is temperature dependent and reversible, with expression at lower temperatures and loss of expression at higher temperatures (Rhoades et al., 2009). This suggests that alternative mechanisms regulating GPL production in the smooth to rough transition are operating. In addition, temperature-dependent transitioning of the smooth to rough phenotype could be a mechanism whereby a GPL expressing environmental strain gains access to abnormal lung airways where higher internal core temperature of the lungs would result in loss of GPL and emergence of the rough virulent phenotype. Our current model of *MABS* pathogenesis is summarized in Figure 1.

With the exception of *MABS* rough strains from chronically infected CF patients contaminating the hospital/surgical or CF clinic environment, the majority of environmental strains have the smooth colony phenotype (Jonsson et al., 2007; Baker et al., 2017). Direct acquisition of a *MABS* rough variant into abnormal lung airways as might occur in a contaminated clinical environment would likely result in a less indolent infection due to the greater virulence of rough variants and stimulation of a robust innate immune response.

## Comparison to Other Clinically Significant Ntm

Other clinically significant NTM may express some form of GPL or exhibit cording, but there are few examples of other NTM which can transition between both phenotypes with such a clear correlation between colonization and invasion. For example, *MAC* expresses serovar-specific GPLs that differ from the non-specific core GPL found in *MABS* through modification by addition of oligosaccharides (Brennan et al., 1981). In contrast to *MABS* GPL, these molecules are immunogenic and are associated with *MAC* virulence (Barrow et al., 1995; Sweet and Schorey, 2006). *M. kansasii* variants may have a smooth colony phenotype associated with expression of characteristic lipids distinct from GPLs (Nataraj et al., 2015). Both *MAC* and *M. kansasii* form microscopic bacterial aggregates in broth culture, but do not exhibit the serpentine cording found in *M. tuberculosis* and *MABS* that is associated with mycobacterial virulence (Tu et al., 2003). In contrast, *M. marinum* does not express GPLs, but does exhibit serpentine cording (Hall-Stoodley et al., 2006). Thus, *MABS* may be viewed as a pathogen which has a unique ability to shift from a ubiquitous environmental saprophyte to an invasive human pathogen – this transformation is apparent upon inspection of the shape of its bacterial colonies growing on nutrient agar.

## The Paradox of Low Virulence and Ineffective Host Response

Perhaps the most puzzling aspect of *MABS* pulmonary infection is that it rarely occurs in immunocompetent individuals with normal lung airways in spite of the ability of this pathogen to invade and replicate in mononuclear phagocytes and non-professional phagocytes (Byrd and Lyons, 1999). Determining the reason(s) why has been impeded by the lack of a suitable mouse model that mimics human lung bronchiectasis. For example, mice with in which the *CFTR* gene has been disrupted have organ specific pathology which is mild in the lung (Clarke et al., 1994). A non-CF mouse model utilizes GM-CSF knock out mice in which a chronic lung infection can be established. In this model, colony forming units in mouse lung persist and gradually decline over a month but then begin to increase at 2 months – at that time the mice have begun to develop bronchiectasis. The limitations of this model are that the mice are immunocompromised, and that bronchiectasis develops late in the course of infection (De Groote et al., 2014). In spite of the current lack of an ideal model, a pattern of bacterial – host interaction is beginning to emerge based on clinical and experimental evidence to date.

At the cellular level, important differences have been reported regarding the intracellular life cycle of *MABS* smooth and rough variants. Since both variants replicate in tissue culture medium assessing growth in macrophages requires extensive washing after infection, incubation with amikacin to kill extracellular bacteria and then removal of amikacin followed by lysis of macrophages and plating lysates for bacterial CFU. Using this model, rough variants replicate intracellularly resulting in lysis of cell monolayers whereas smooth variants persist and/or decline within cells, but do not destroy cell monolayers in the time frames studied in these experiments (Byrd and Lyons, 1999; Howard et al., 2006; Greendyke and Byrd, 2008; Nessar et al., 2011). Recent studies have demonstrated differences in intracellular behavior comparing smooth and rough variants which may account for these findings. Smooth variants have been found to reside in phagosomes in which they are surrounded by electron translucent zone representing cell wall GPL. They are typically present as a single organism which is likely due to the fact that they are easily dispersed into single cell suspensions prior to infecting cells. In contrast, rough variants clump in a manner similar to *M. tuberculosis* and are difficult to get into single cells suspension, thus they are usually present as two or more bacteria per phagosome (Byrd and Lyons, 1999; Roux et al., 2016). Consistent with the lack of destruction of macrophage monolayers infected with smooth variants (Nessar et al., 2011) is the finding that smooth variants are poor inducers of apoptosis and autophagy in contrast to rough variants which end up in phagolysosomes but nonetheless replicate intracellularly and induce apoptosis (Roux et al., 2016). In fact, uptake of large clumps of *MABS* rough variants leads to rapid dissolution of macrophages and emergence of *MABS* cords (Brambilla et al., 2016). There is evidence that smooth variants cause disruption of the phagosomal membrane allowing for direct cytosolic contact (Roux et al., 2016). Thus, as a mechanism of apoptosis inhibition, polar GPLs found on the surface of *MABS* smooth variants (Lopez-Marin et al., 1994; Howard et al., 2006) have access to cytosolic contents and have been demonstrated to interact with mitochondrial cyclophilin D, a component of the mitochondrial permeability transition pore (MPTP) to stabilize the pore and inhibit apoptosis (Whang et al., 2017).

*MABS* has an ESX-4 type VII secretion system encoded for by five genes. A gene in this locus, *eccB4* was found to be necessary for low level replication (approximately 0.5 log over 5 days), but not persistence, of the smooth *MABS subspecies massiliense* 43S strain in *Acanthamoeba castellani* and J774.2 mouse macrophages. This gene was also found to be necessary for prevention of phagosome acidification and for causing phagosome rupture (Laencina et al., 2018). Other studies have found that under stringent conditions to prevent extracellular replication in tissue culture media, colony forming units of *MABS* smooth variants in human monocyte-derived macrophage monolayers have limited replicative capacity over a 5 day period and do not persist in the lungs of infected SCID mice (Byrd and Lyons, 1999; Howard et al., 2006). These latter findings raise the question of the significance of the slight loss of replicative ability in the *eccB4* deletion mutant. The question of the effect of expression of the genes of the ESX-4 type VII type secretion system by the more virulent rough *MABS* phenotype is unexplored although as noted, another study did not report phagosome disruption but rather trafficking of rough variants through the autophagic pathway ending up in phagolysosomes where they replicate and rapidly induce apoptosis (Roux et al., 2016). It remains unclear why rough variants, which are able to arise from the smooth variant used in this study (Ripoll et al., 2009), would not also rupture the phagosome. Finally, as would be expected for an intracellular pathogen establishing cytosolic contact and activating the inflammasome, this study found that IL1β release was increased by cells infected with wild type bacteria. There was a significant decrease in IL1β release from cells infected with the *eccB4* deletion mutant. Since inflammasome activation results in cell death via pyroptosis (Sharma and Kanneganti, 2016), and human macrophage monolayers infected with *MABS* smooth variants typically remain intact and viable throughout the course of infection, this discrepancy remains unexplained. It may be that GPL-mediated inhibition of macrophage apoptosis counteracts the effect of inflammasome activation (Whang et al., 2017).

One important function of GPL is that it prevents bacteria to bacteria interaction of underlying surface molecules such as trehalose polyphleates which may play a role in the cording exhibited by rough variants (Llorens-Fons et al., 2017). With loss of GPL, rough variants taken up by macrophages rapidly induce apoptosis (Roux et al., 2016) and demonstrate rapid cell–cell spread via serpentine cord formation (Byrd and Lyons, 1999). The fact that serpentine cording is an important virulence determinant of rough *MABS* strains was demonstrated using a rough deletion mutant lacking a gene coding for a dehydratase necessary for cord formation. This mutant was found to be markedly attenuated for virulence in the zebrafish model (Halloum et al., 2016). A comparison of the behavior of *MABS* rough and smooth variants to the virulent *M. tuberculosis* strains H37Rv/Erdman, and the avirulent strain H37Ra shows similar behavior in a fibroblast microcolony assay which we described (Byrd et al., 1998; Byrd and Lyons, 1999). The differences relate to the addition of extracellular acting aminoglycoside antibiotics. Both the *MABS* rough variant and H37Rv/Erdman demonstrate elongated, corded microcolonies within the plane of the agar-overlaid monolayers, while the *MABS* smooth variant and H37Ra demonstrate significantly smaller, rounded microcolony morphology. Importantly, the addition of streptomycin does not prevent formation of H37Rv/Erdman microcolonies, presumably due to a mechanism of direct cell–cell spread wherein *M. tuberculosis* avoids exposure to the extracellular environment. In contrast, addition of amikacin to fibroblast monolayers infected with the *MABS* rough variant prevents the formation of corded microcolonies. This suggests that *M. tuberculosis* is host adapted to favor replication within the intracellular environment while *MABS* may be viewed as an environmental saprophyte that has not quite made the transition to an intracellular lifestyle.

In spite of the ability to replicate extracellularly and form microabscesses in the zebrafish model of infection, *MABS* pulmonary infection does not occur in immunocompetent humans (or mice) with normal lung airways. Why this is so is a central question in terms of *MABS* pathogenesis. TLR2 has been found to be important in innate immune recognition and signaling in response to *MABS*. PIMs exposed on the surface of rough variants interact directly with TLR2 on human macrophages and epithelial cells to promote release of TNFα and IL-8, respectively, whereas PIMs are “masked” by GPL on the surface of smooth variants which are not recognized by TLR2 (Rhoades et al., 2009; Davidson et al., 2011). Both TNFα and IL-8 have both been found to be important for control of infection by *MABS* rough variants in the zebrafish model of infection (Bernut et al., 2016a). On the other hand, human neutrophils have been found to be less effective at killing *MABS* than killing *S. aureus*, and dead and dying neutrophils have been found to enhance biofilm formation by smooth variants (Malcolm et al., 2013). Thus in abnormal lung airways with impaired mucociliary clearance and *MABS* smooth variant biofilm formation, ongoing inflammation with IL-8 mediated neutrophil recruitment may promote biofilm persistence. This may have particular relevance in patients with CF who are often co-infected with multiple pulmonary pathogens and in whom chronic lung airway inflammation dominated by the presence of neutrophils is felt to be central to disease pathogenesis (Cantin et al., 2015). Another aspect of the innate immune response relates to single nucleotide polymorphisms (SNPs) that alter host responses to infectious agents. TLR2 signaling in response to mycobacterial lipopeptides depends upon formation of TLR2/TLR1 heterodimers at the cell surface. SNPs that alter the function of either TLR2 and/or TLR1 may thus affect innate immune responses to mycobacteria. We have reported that a well described SNP, TLR1 SNP I602S, is present in the respiratory epithelial cell line CFBE41o-, which was derived from the bronchus of a patient with CF and immortalized with SV40. This cell line is hyporesponsive to TLR2/TLR1 receptor agonist and the rough *MABS* 390R strain. This SNP is likely to be present in the CF patient population (Kempaiah et al., 2013). Paradoxically the presence of this SNP is protective against infection with *Mycobacterium leprae* (Johnson et al., 2007). An unanswered question is how the presence of this SNP affects susceptibility to NTM infection in both CF and non-CF patients.

In terms of the cell-mediated immune response, as would be predicted, anti-TNFα inhibitor therapy has been associated with disseminated NTM infection, including *MABS* (Winthrop et al., 2009). Th1 CD4+ T cell responses are also important for control of infection with NTM. It is established that patients with advanced HIV and low CD4+ T cell counts are susceptible to infection with *MAC*, and cases of disseminated *MABS* infection have been reported in these patients as well (Tan et al., 2010). The importance of IFNγ in control of *MABS* infection is highlighted in a recent case-control study from Thailand in which *MABS* was found to be the most common NTM clinical isolate, and the presence of anti-IFNγ autoantibody was strongly associated with disseminated infection (Phoompoung et al., 2017). Since defects in cell-mediated immunity have not been identified in the majority of non-CF patients with chronic *MABS* lung infection, it remains unclear why patients with underlying lung airway abnormalities such as bronchiectasis are uniquely susceptible to infection, and why an effective cell-mediated immune response does not develop in response to pulmonary infection.

## A Limited Antimicrobial Armamentarium

In spite of its low virulence compared to pathogens such as *M. tuberculosis*, *MABS* infection of the lung is the most difficult NTM to treat, resembling multi-drug resistant tuberculosis. Numerous resistance mechanisms in *MABS* have been identified that limit the number of available antibiotics in comparison to infections caused by other NTM (Brown-Elliott et al., 2012; Nessar et al., 2012; Luthra et al., 2018). Of the limited antibiotics used to treat *MABS* infection most are bacteriostatic and not bactericidal for both intra- and extracellular bacteria *in vitro* (Greendyke and Byrd, 2008; Maurer et al., 2014). *MABS* biofilm formation has been described for GPL-expressing smooth variants, and biofilm consisting primarily of smooth variants has been found in the lung airways of explanted lungs from CF patients prior to lung transplantation (Qvist et al., 2015). Under certain *in vitro* culture conditions *MABS* rough variants grow as biofilms as well (Clary et al., 2018). Biofilms formed by both smooth and rough *MABS* variants are relatively resistant to antibiotics when compared to planktonic bacteria (Greendyke and Byrd, 2008; Clary et al., 2018). The bacteriostatic activity of currently used antibiotics against intracellular *MABS*, and the relative antibiotic resistance of *MABS* biofilms in abnormal lung airways make eradication of *MABS* from the lung extremely difficult.

Of the antibiotics used to treat *MABS* infection, macrolides are felt to be the cornerstone of therapy. Importantly, there are differences in macrolide susceptibility among the different *MABS* subspecies based on the presence and functional status of the *erythromycin ribosomal methylation gene 41* (*erm41*). The *erm41* gene encodes for an enzyme that confers intrinsic, inducible resistance in *MABS* (Nash et al., 2009). In *MABS* strains with a functional *erm41* gene, clarithromycin may initially appear to be active; however, resistance may develop in the time frame of 3–14 days, the Clinical and Laboratory Standard Institute (CLSI) recommendation for length of incubation (Nash et al., 2009; Koh et al., 2011; CLSI, 2011). PCR is now being used to identify *MABS* subspecies and *erm41* gene status (Shallom et al., 2015).

Broth microdilution with determination of minimum inhibitory concentration (MIC) is recommended as the gold standard for NTM antibiotic susceptibility testing. Due to the relative rarity of *MABS* infections in the past, correlating *in vitro* modeling and susceptibility testing with effective clinical response has been limited. In fact, tigecycline, a glycine antibiotic commonly used in treatment of *MABS* infection, currently lacks MIC interpretation from CLSI (Brown-Elliott et al., 2012). When *MABS* infection involves sites such as the CNS and bone, it is important to consider pharmacokinetic properties to maximize antibiotics exposure at the target site (Landersdorfer et al., 2009; Nau et al., 2010). Dosing regimens should be individualized to maximize drug exposure at the target site while limiting potential side-effects. The use of inhaled aminoglycosides for pulmonary *MABS* infection is one strategy to maximize the concentration at the active site while decreasing the likelihood of side-effects. Several small trials of inhaled amikacin for the treatment of NTM, specifically *MABS* and *MAC*, have had positive results including negative culture conversion and improvement seen on lung imaging (Olivier et al., 2014; Yagi et al., 2017). Even though serum concentrations are lower with inhaled amikacin it is not completely without risk. In one study, ototoxicity occurred at a relatively high rate (Olivier et al., 2014). Inhaled liposomal amikacin may be a further advancement in localized therapy for *MABS*. The liposome capsule penetrates biofilms and is also taken up by macrophages within the lung, thus delivering amikacin directly to the site of infection (Zhang et al., 2018). It should be noted that a Phase 2 trial of inhaled liposomal amikacin in addition to standard therapy failed to meet its primary endpoint for both *MABS* and *MAC* but did show improvement in culture clearance and 6-minute walk test (Olivier et al., 2017). Inhaled liposomal amikacin has recently been approved in the United States for the treatment of refractory *MAC* following a Phase 3 trial that demonstrated an increase in sputum clearance at 6 months (Griffith et al., 2018). More data about the use specifically in *MABS* is still needed. It is apparent that development of new drugs specifically targeting *MABS* infection should be a high priority and drug discovery efforts are underway utilizing novel high throughput screening platforms (Gupta et al., 2017). There are currently two new antibiotics designed and approved for alternative infectious indications which may add to the *MABS* antimicrobial armamentarium and are worth mentioning. The first antibiotic is an FDA-approved agent named tedizolid. Compared to the related antibiotic linezolid which is generally active against *MABS*, tedizolid has a higher ribosomal binding affinity that allows for lower effective serum concentration and once daily dosing (Burdette and Trotman, 2015). *In vitro* testing of NTM isolates shows promise as MIC values are 1–8 times lower than that of linezolid (Brown-Elliott and Wallace, 2017). Importantly, tedizolid has a lower incidence of side-effects and drug interactions as compared to linezolid (Burdette and Trotman, 2015). There is little data on long-term use of tedizolid that has been reported, but overall it appears to be well tolerated (Nigo et al., 2018). The second antibiotic is avibactam, a non-β-lactam β-lactamase inhibitor commercially available as a coformulation with ceftazidime, which can inactivate the *MABS* β-lactmase Bla_mab_. When avibactam is combined with β-lactams, this combination may have enhanced activity for the treatment of *MABS* infection (Kaushik et al., 2017; Le Run et al., 2018).

## Future Directions

*Mycobacterium abscessus* (*MABS*) has emerged as a significant infectious disease threat and warrants the designation of “shapeshifter of the mycobacterial world.” Its ability to exist as a GPL-expressing environmental saprophyte forming biofilms along with the ability to “unmask” itself within the human host and display virulence properties such as serpentine cording is unprecedented for a bacterial pathogen. The ability of evolving clones to spread among clinical environments foreshadows an increasing incidence of nosocomial infections. The fact that many strains are multidrug resistant and that the *MABS* subspecies differ in terms of their inherent antimicrobial susceptibility creates an enormous challenge, particularly since patients often require months of therapy to achieve cure. There is an urgent need for better models that mimic human pulmonary infection to better understand disease pathogenesis and test new compounds for antimicrobial activity.

## Author Contributions

KR contributed ideas and expertise, and drafted the section related to antimicrobial therapy. TFB conceived and wrote the final manuscript.

## Conflict of Interest Statement

The authors declare that the research was conducted in the absence of any commercial or financial relationships that could be construed as a potential conflict of interest.
